# The global Minmax *k*-means algorithm

**DOI:** 10.1186/s40064-016-3329-4

**Published:** 2016-09-27

**Authors:** Xiaoyan Wang, Yanping Bai

**Affiliations:** 1 School of Information and Communication Engineering, North University of China, Taiyuan, 030051 People’s Republic of China; 2 School of Science, North University of China, Taiyuan, 030051 People’s Republic of China

**Keywords:** *k*-Means, Clustering, MinMax *k*-means, Global *k*-means

## Abstract

The global *k*-means algorithm is an incremental approach to clustering that dynamically adds one cluster center at a time through a deterministic global search procedure from suitable initial positions, and employs *k*-means to minimize the sum of the intra-cluster variances. However the global *k*-means algorithm sometimes results singleton clusters and the initial positions sometimes are bad, after a bad initialization, poor local optimal can be easily obtained by *k*-means algorithm. In this paper, we modified the global *k*-means algorithm to eliminate the singleton clusters at first, and then we apply MinMax *k*-means clustering error method to global *k*-means algorithm to overcome the effect of bad initialization, proposed the global Minmax *k*-means algorithm. The proposed clustering method is tested on some popular data sets and compared to the *k*-means algorithm, the global *k*-means algorithm and the MinMax *k*-means algorithm. The experiment results show our proposed algorithm outperforms other algorithms mentioned in the paper.

## Background

Clustering is one of classic problems in pattern recognition, image processing, machine learning and statistics (Xu and Wunsch 2005; Jain 2010; Berkhin 2006). Its aim is to partition a collection of patterns into disjoint clusters, such that patterns in the same cluster are similar, however patterns belonging to two different clusters are dissimilar.

One of the most popular clustering method is *k*-means algorithm, where clusters are identified by minimizing the clustering error. Despite its popularity, the *k*-means algorithm is sensitive to the choice of initial starting conditions (Celebi et al. 2013; Peña et al. 1999; Celebi and Kingravi 2012, 2014). To deal with this problem, the global *k*-means algorithm has been proposed (Likas et al. 2003), and then some of its modifications (Bagirov 2008; Bagirov et al. 2011) are proposed. Even an extension to kernel space has been developed (Tzortzis and Likas 2008, 2009). A fuzzy clustering version is also available (Zang et al. 2014). All of these are incremental approaches that start from one cluster and at each step a new cluster is deterministically added to the solution according to an appropriate criterion. Using this method also can learn the number of data clusters (Kalogeratos and Likas 2012). Although the global k-means algorithm is deterministic and often performs well, but sometimes the new cluster center may be a outlier, then it may arise that some of the clusters just have single point, the result is awful. Another way to avoid the choice of initial starting conditions is to use the multi restarting *k*-means algorithm (Murty et al. 1999; Arthur and Vassilvitskii 2007; Banerjee and Ghosh 2004). A new version of this method is the MinMax *k*-means clustering algorithm (Tzortzis and Likas 2014), which starts from a randomly picked set of cluster centers and tries to minimize the maximum intra-cluster error. Its application (Eslamnezhad and Varjani 2014) shows that the algorithm is efficient in intrusion detection.

In this paper, a new version of modified global *k*-means algorithms is proposed in order to avoid the singleton clusters. In addition, the initial positions chosen by the global *k*-means algorithms sometimes are bad, after a bad initialization, poor local optimal can be easily obtained by *k*-means algorithm. Therefore we employ the MinMax *k*-means clustering error method instead of *k*-means clustering error in global *k*-means algorithm to tackle this problem, obtain a deterministic algorithm called the global Minmax *k*-means algorithm. We do loads of experiments on different data sets, the results show that our proposed algorithm is better than other algorithms which referred in the paper.

The rest of paper is organized as follows. We briefly describe the *k*-means, the global *k*-means and the MinMax *k*-means algorithms in “Preliminaries” section. In “The proposed algorithm” section we proposed our algorithms. Experimental evaluation is presented in “Experiment evaluation” section. Finally “Conclusions” section conclude our work.

## Preliminaries

### *k*-Means algorithm

Given a data set $X=\{x_1,x_2,\ldots ,x_N\}, x_n\in R^d (n=1,2,\ldots ,N)$. We aim to partition this data set into *M* disjoint clusters $C_1,C_2,\ldots ,C_M$, such that a clustering criterion is optimized. Usually, the clustering criterion is the sum of the squared Euclidean distances between each data point $x_n$ and the cluster center $m_k$ that $x_n$ belongs to. This kind of criterion is called clustering error and depends on the cluster centers $m_1,m_2,\ldots ,m_k$:

1 $$\begin{aligned} E\left( m_1,m_2,\ldots ,m_M\right) =\sum \limits _{i=1}^{N}\sum \limits _{k=1}^{M}I\left( x_i\in C_k\right) \Vert x_i-m_k\Vert ^2, \end{aligned}$$

where

$$\begin{aligned} I(X)=\left\{ \begin{array}{ll} 1,&{}\quad X{\text { is true}},\\ 0,&{}\quad {\text {Otherwise}}.\end{array}\right. \end{aligned}$$

Generally, we call $\sum \nolimits _{k=1}^{M}I(x_i\in C_k)\Vert x_i-m_k\Vert ^2$ intra-cluster error(variance). Obviously, clustering error is the sum of intra-cluster error. Therefore, we use $E_{sum}$ instead of $E(m_1,m_2,\ldots ,m_M)$ in briefly, i.e. $E_{sum}=E(m_1,m_2,\ldots ,m_M)$.

The *k*-means algorithm finds locally optimal solutions with respect to the clustering error. The main disadvantage of the method is its sensitivity to initial position of the cluster center.

### The global *k*-means algorithm

To deal with the initialization problem, the global *k*-means has been proposed, which is an incremental deterministic algorithm that employs *k*-means as a local search procedure. This algorithm obtains optimal or near-optimal solutions in terms of clustering error.

In order to solve a clustering problem with *M* clusters, Likas et al. (2003) provided the proceeds as follows. The algorithm starts with one cluster $(k=1)$ and find its optimal position which corresponds to the data set centroid. To solve the problem with two clusters $(k=2)$ they run *k*-means algorithm *N* (*N* is the size of the data set) times, each time starting with the following initial positions of the cluster centers: the first cluster center is always placed at the optimal position for the problem with $k=1$, and the other at execution *n* is placed at the position of the data point $x_n(n=1,2,\ldots ,N)$. The solution with the lowest cluster error is kept as the solution of the 2-clustering problem. In general, let $(m_1^*,m_2^*,\ldots ,m_k^*)$ denote the final solution for *k*-clustering problem. Once they find the solution for the $(k-1)$-clustering problem, they try to find the solution of the *k*-clustering problem as follows: they perform *N* executions of the *k*-means algorithm with $(m_1^*,m_2^*,\ldots ,m_{(k-1)}^*,x_n)$ as initial cluster centers for the $n\hbox {th}$ run, and keep the solution resulting in the lowest clustering error. By proceeding in the above fashion they finally obtain a solution with *M* clusters and also found solutions for all *k*-clustering problems with $k<M$.

This version of the algorithm is not applicable for clustering on middle sized and large data sets. Two modifications were proposed to reduce the complexity (Likas et al. 2003), and we interest in the first procedure. Let $d_{k-1}^j$ is the squared distance between $x_j$ and the closest center among the $k-1$ cluster centers obtained so far. In order to find the starting point for the *k*th cluster center, for each $x_n\in R^d,n=1,2,\ldots ,N$ we compute $b_n$ as follows.

2 $$\begin{aligned} b_n=\sum \limits _{i=1}^{N}\max \left( d_{k-1}^j-\Vert x_n-x_j\Vert ^2,0\right) , \end{aligned}$$

The quantity $b_n$ measures the reduction in the error measure obtained by inserting a new cluster center at point $x_n$. It is clear that a data point $x_n\in R^d$ with the largest value of the $b_n$ is the best candidate to be a starting point for the *k*th cluster center. Therefore, we compute $i=\arg \max \nolimits _{n} b_n$ and find the data point $x_n\in R^d$ such that $b_n=i$. This data point is selected as a starting point for the *k*th cluster center.

### The MinMax *k*-means algorithm

As we known, in the *k*-means algorithm, we minimize the clustering error. Instead of this method, the MinMax *k*-means algorithm minimizes the maximum intra-cluster error

3 $$\begin{aligned} E_{\max }=\max _{1\le k\le M}\sum \limits _{i=1}^{N}I(x_i\in C_k)\Vert x_i-m_k\Vert ^2, \end{aligned}$$

where $m_k,I(x)$ are defined as (1).

Since directly minimizing the maximum intra-cluster variance $E_{\max }$ is difficult, a relaxed maximum variance objective was proposed (Tzortzis and Likas 2014). They constructed a weighted formulation $E_w$ of the sum of the intra-cluster variances (4)

4 $$\begin{aligned} \begin{array}{ll} E_w=\sum \limits _{k=1}^{M} w_k^p\sum \limits _{i=1}^{N}I\left( x_i\in C_k\right) \Vert x_i-m_k\Vert ^2,\\ w_k\ge 0,\sum \limits _{k=1}^{M}w_k=1, \quad 0\le p\le 1. \end{array} \end{aligned}$$

where the *p* exponent is a constant. The greater(smaller) the *p* value is, the less(more) similar the weight values become, as relative differences of the variances among the clusters are enhanced(suppressed).

Now, all clusters contribute to the objective, according to different degrees regulated by the $w_k$ values. It is clear that the more a cluster contributes (higher weight), the more intensely its variance will be minimized. So $w_k$ are calculated by formula (5)

5 $$\begin{aligned} w_k=v_k^{1\diagup (1-p)}\Big /\sum \limits _{k'=1}^{M} v_{k'}^{1\diagup (1-p)}, \quad {\text {where}}\, v_k=\sum \limits _{i=1}^{N}I(x_i\in C_k)\Vert x_i-m_k\Vert ^2. \end{aligned}$$

To enhance the stability of the MinMax *k*-means algorithm, a memory effect could be added to the weights:

6 $$\begin{aligned} w_k^{(t)}=\beta w_k^{t-1}+(1-\beta )\left( v_k^{1\diagup (1-p)}\Big / \sum \limits _{k'=1}^{M} v_{k'}^{1\diagup (1-p)}\right) ,\quad 0\le \beta \le 1. \end{aligned}$$

## The proposed algorithm

### The modified global *k*-means algorithm

As we known, the global *k*-means algorithm may obtain singleton clusters if the initial centers are outliers. To avoid this, we propose the Modified global *k*-means algorithm.

Algorithm 1: The Modified global *k*-means Algorithm 1.

Step 1 (Initialization) Compute the centroid $m_1$ of the data set *X*:

7 $$\begin{aligned} m_1=\frac{1}{N}\sum \limits _{i=1}^{N}x_i,\,x_i\in X,\quad i=1,2,\ldots ,N. \end{aligned}$$

and $$k=1$$;

Step 2 (Stopping criterion) Set $k=k+1$. If $k>M$, then stop;

Step 3 Take the centers $m_1,m_2,\ldots ,m_{k-1}$ from the previous iteration and consider each point $x_i$ of *X* as a starting point for the *k*th cluster center, thus obtain *N* initial solutions with *k* points $(m_1,m_2,\ldots ,m_{k-1},x_i)$;

Step 4 Apply the *k*-means algorithm to each of them; keep the best *k*-partition obtained and its centers $y_1,y_2,\ldots ,y_k$;

Step 5 (Detect the singleton clusters) If the obtained clusters exist singleton cluster, then delete the point $y_k$ in candidate initial center *X*, and go to step 3, else go to step 6;

Step 6 Set $m_i=y_i,\,i=1,2,\ldots ,k\,$ and go to step2.

Due to high computational cost of the global *k*-means algorithm, we propose the fast algorithm. It is based on the idea as the fast global *k*-means variant proposed in Peña et al. (1999).

Algorithm 2: The Modified global *k*-means Algorithm 2.

The steps 1, 2, 6 are same to the Algorithm 1.

Steps 3, 4, 5 is modified as follows:

Step 3′ Take the centers $m_1,m_2,\ldots ,m_{k-1}$ from the previous iteration and consider each point $x_i$ of *X* as a starting point for the *k*th cluster center, then calculate $b_i$ using Eq. (2), choose the corresponding starting point of maximum $b_i$ as the best solution;

Step 4′ Apply the *k*-means algorithm to the best solution; keep the best *k*-partition obtained and its centers $y_1,y_2,\ldots ,y_k$;

Step 5′ (Detect the singleton clusters) If the obtained clusters exist singleton cluster $b_i$, then let $b_i=0$, and go to step 3, else go to step 6;

In our numerical experiments we use Algorithm 2.

Our proposed algorithm based on realistic data set. The data set includes 41 students scores, and each student has 11 subjects grades. When we use the global *k*-means algorithm to cluster students according to their scores of subjects, the output is bad. The comparisons between the global *k*-means algorithm and the modified global *k*-means algorithm in Table 1.

**Table 1:** Comparative results

Method	Clusters	$E_{sum}$	Number of each cluster
Global *k*-means	4	1.0e+04 $\times$ 4.9175	(25, 14, 1, 1)
Modified global *k*-means	4	1.0e+04 $\times$ 4.0718	(12, 14, 13, 2)

Table 1 shows when we partition the data for four clusters, there are two clusters just include one element in the global *k*-means algorithm, i.e. there are two singleton clusters in the global *k*-means algorithm. We also find that the $E_{sum}$ of modified global *k*-means is more lower than that of global *k*-means.

### The global Minmax *k*-means algorithm

**Fig. 1:**
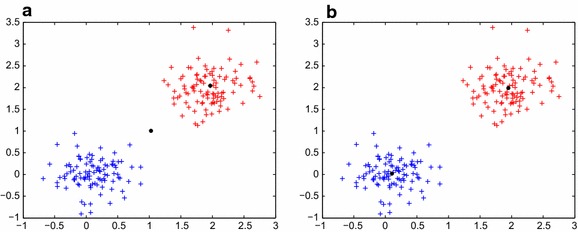
Example **a** is the initial point for $k=2$ using the global algorithm, and it’s clear that it is a bad initial point. Example **b** shows a better initial point

The global *k*-means algorithm is a deterministic global search procedure from suitable initial positions, but the initial positions sometimes are poor. An example is illustrated in Fig. 1. The MinMax *k*-means algorithm was verified effective and robust over bad initializations (Murty et al. 1999), but its not deterministic, it needs multiple restarts. So we combine the global *k*-means algorithm and the MinMax *k*-means algorithm, i.e. we apply MinMax *k*-means clustering error method to the global *k*-means algorithm, then we get a deterministic algorithm called the global Minmax *k*-means algorithm.

The global Minmax *k*-means algorithm is an incremental approach to clustering that dynamically adds one cluster center at a time through a deterministic global search procedure from suitable positions like the global *k*-means algorithm, and this procedure was introduced in preliminaries. After choose the initial center, we employ the MinMax *k*-means method to minimize the maximum intra-cluster variances. The MinMax *k*-means algorithm was described in preliminaries. The whole method of the proposed algorithm is illustrated as Algorithm 3.

Algorithm 3: The global Minmax *k*-means algorithm.

Step 1 (Initialization) Compute the centroid $m_1$ of the set *X*, using (7).

Step 2 (Stopping criterion) Set $k=k+1$. If $k>M$, then stop;

Step 3 Take the centers $m_1,m_2,\ldots ,m_{k-1}$ from the previous iteration and consider each point $x_i$ of *X* as a starting point for the *k*th cluster center, thus obtaining *N* initial solutions with *k* points $(m_1,m_2,\ldots ,m_{k-1},x_i)$;

Step 4 Apply the MinMax *k*-means algorithm to each of them; keep the best *k*-partition obtained and its centers $y_1,y_2,\ldots ,y_k$;

Step 5 (Detect the singleton clusters) If the obtained clusters exist singleton cluster, then the candidate initial center delete the point $y_k$, and go to step 3, else go to step 6;

Step 6 Set $m_i=y_i,\,i=1,2,\ldots ,k\,$ and go to step 2.

## Experiment evaluation

In the following subsections we provide extensive experimental results comparing the global Minmax *k*-means algorithm with *k*-means algorithm, the global *k*-means algorithm and the Minmax *k*-means algorithm. In the experiments, the results of *k*-means algorithm and the MinMax *k*-means algorithm are the average of $$E_{max}$$$$E_{sum}$$ defined by (3) (1) , which restart 100 times. For the MinMax *k*-means algorithm and the global Minmax *k*-means algorithm, some additional parameters ($$\beta ,p$$) must be fixed prior to execution. In Tzortzis and Likas (2014), there gives a practical framework that extends the MinMax *k*-means to automatically adapt the exponent *p* to the data set. It begins with a small *p* ($$p_{init}$$) that after each iteration is increased by $$p_{step}$$, until a maximum value *p* ($$p_{max}$$) is attained. As the method, we should decide parameter $$p_{init}$$, $$p_{max}$$ and $$p_{step}$$ at first. We set $$p_{init}=0,\,p_{step}=0.01$$ and using *p* instead of $$p_{max}$$ for all MinMax *k*-means and global Minmax *k*-means algorithm experiments. In Tables 2, 3 and 8, we did not mark the value of parameter *p*, since for different *p* has the same result.

**Table 2:** Comparative results on $S_1$ data set

Method	$E_{max}$	$E_{sum}$
*k*-Means	28.4856	96.6753
Global *k*-means	25.3388	93.7457
MinMax *k*-means ($\beta =0.3$)	25.3388	93.7457
MinMax *k*-means ($\beta =0.1$)	25.3388	93.7457
MinMax *k*-means ($\beta =0$)	25.3388	93.7457
Global Minmax *k*-means ($\beta =0.3$)	25.3388	93.7457
Global Minmax *k*-means ($\beta =0.1$)	25.3388	93.7457
Global Minmax *k*-means ($\beta =0$)	25.3388	93.7457

**Table 3:** Comparative results on $S_2$ data set

Method	$E_{max}$	$E_{sum}$
*k*-Means	52.0518	197.4535
Global *k*-means	52.0518	197.4535
MinMax *k*-means ($\beta =0.3$)	52.0518	197.4535
MinMax *k*-means ($\beta =0.1$)	52.0518	197.4535
MinMax *k*-means ($\beta =0$)	52.0518	197.4535
Global Minmax *k*-means ($\beta =0.3$)	52.0518	197.4535
Global Minmax *k*-means ($\beta =0.1$)	52.0518	197.4535
Global Minmax *k*-means ($\beta =0$)	52.0518	197.4535

### Synthetic data sets

**Fig. 2:**
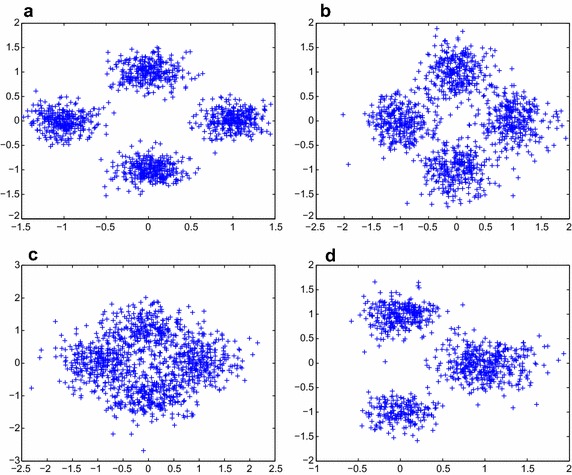
The sketch of four typical synthetic data sets: **a** $S_1$, **b** $S_2$, **c** $S_3$, **d** $S_4$

Four typical synthetic data sets $S_1,S_2,S_3,S_4$ are tested in this section, as in Fang et al. (2013). Typically, they are generated from a mixture of four or three bivariate Gaussian distribution on the plane coordinate system. Thus a cluster takes the form of a Gaussian distribution. Particularly, all the Gaussian distribution have the covariance matrices have the form of $\sigma ^{2}I$, where $\sigma$ is the standard variance. For the first three data sets, four Gaussian distributions, all with 300 sample points, are all located at $(-1,0),(1,0),(0,1)$ and $(0,-1)$, respectively, and their standard variances $\sigma$ keep the same, but vary with the data sets. Actually, $\sigma$ takes the values of 0.2, 0.3, 0.4 for $S_1,S_2,S_3$, respectively. In this way, the degree of overlap among the clusters increases considerably from $S_1$ to $S_3$ and therefore the corresponding classification problem becomes more complicated. As for $S_4$, we give three Gaussian distributions located at (1, 0), (0, 1) and $(0,-1)$, with 400, 300, 200 sample points, respectively. Therefore, $S_4$ represents the asymmetric situation where the clusters do not take the same shape, and also with different number of sample points. The data sets are shown in Fig. 2 respectively.

### Real-world data sets

Coil-20 is a data set (Nene et al. 1996), which contains 72 images taken from different angels for each of the 20 included objects. We used three subsets Coil15, Coil8, Coil19, with images from 15, 18 and 19 objects, respectively, as the data set in Tzortzis and Likas (2014). The data set includes 216 instances and each of the data has 1000 features.

Iris(UCI) (Frank and Asuncion 2010) is a famous data set which created by R.A. Fisher. There are 150 instances and 50 in each of three classes. Each data has four predictive attributes.

Seeds(UCI) (Frank and Asuncion 2010) is composed of 210 records that extract from three different varieties of wheat. The number of each grain is equal and each grain is described by seven features.

Yeast(UCI) (Frank and Asuncion 2010) includes 1484 instances about the cellular localization sites of proteins and eight attributes. Proteins belong to ten categories. Five of the classes are extremely under represented and are not considered in our evaluation. The data set is unbalanced.

Pendigits(UCI) (Frank and Asuncion 2010) includes 10,992 instances of handwritten digits (0–9) from the UCI repository (Eslamnezhad and Varjani 2014), and 16 attributes. The data set is almost balanced.

User Knowledge Modeling (UCI) (Frank and Asuncion 2010) is about the students’ knowledge status about the subject of Electrical DC Machines. User Knowledge Modeling includes 403 instances with 6-dimensional space. The data set is unbalanced. The students are assessed four levels.

In the experiment, the sample data of Iris, Seeds and Pendigits data set will be normalized using z-score method firstly and the algorithm will be implemented on the normalized data.

A summary of the data sets is provided in Table 4.

### Performance analysis

The comparison of the algorithms across the various data sets is shown in Tables 2, 3, 4, 5, 6, 7, 8, 9, 10, 11 and 12, except Table 6. In Tables 2, 3, 4, 5, 6, 7, 8, 9, 10, 11 and 12, first, we find that the global Minmax *k*-means algorithm attains better $E_{max}$ than *k*-means algorithm and global algorithm, and in most of cases it better than the MinMax *k*-means algorithm, sometimes equal to the MinMax *k*-means algorithm. Second, the proposed method outperforms *k*-means algorithm for all the metrics reported in Tables 2, 3, 4, 5, 6, 7, 8, 9, 10, 11 and 12 except in Table 3, which get the same result for all algorithms. Third, the global Minmax *k*-means algorithm can reach the lowest $E_{sum}$, except in Tables 7 and 10. As our method employs both the global *k*-means and the MinMax *k*-means algorithm, it perform better than each of the algorithm or sometimes attain the same effect. In Tables 4, 5, 11 and 12, our proposed method attain both the lowest $E_{max}$ and the $E_{sum}$. In Table 11, although global *k*-means reach the lowest $E_{sum}$ too, but when it attain the point, its $E_{sum}$ is bigger than ours. In Tables 4 and 5, the MinMax *k*-means algorithm also can reach the lowest $E_{max}$, but it can not attain the lowest $E_{sum}$. In Tables 7 and 10, the proposed method can not result the lowest $E_{sum}$, but just the method can attain the lowest $E_{max}$. In Tables 2 and 9, all algorithms except *k*-means make the equal effect. In Table 8, MinMax *k*-means and global Minmax *k*-means algorithm run in the same result. They are better than *k*-means and global *k*-means.

**Table 4:** Comparative results on $S_3$ data set

Method	$E_{max}$	$E_{sum}$
*k*-Means	90.8431	329.4181
Global *k*-means	90.8431	*329*.*4133*
MinMax *k*-means ($p=0.5,\beta =0.3$)	*87*.*1170*	329.6677
MinMax *k*-means ($p=0.5,\beta =0.1$)	*87*.*1170*	329.6677
MinMax *k*-means ($p=0.5,\beta =0$)	*87*.*1170*	329.6352
MinMax *k*-means ($p=0.3,\beta =0.3$)	88.4824	329.4766
MinMax *k*-means ($p=0.3,\beta =0.1$)	88.4824	329.4766
MinMax *k*-means ($p=0.3,\beta =0$)	88.5052	329.4761
MinMax *k*-means ($p=0.1,\beta =0.3$)	89.6205	329.4349
MinMax *k*-means ($p=0.1,\beta =0.1$)	89.5976	329.4351
MinMax *k*-means ($p=0.1,\beta =0$)	89.6203	329.4346
MinMax *k*-means ($p=0,\beta =0$)	90.8430	329.4181
Global Minmax *k*-means ($p=0.5,\beta =0.3$)	*87*.*1170*	329.6677
Global Minmax *k*-means ($p=0.5,\beta =0.1$)	*87*.*1170*	329.6677
Global Minmax *k*-means ($p=0.5,\beta =0$)	*87*.*1170*	329.6352
Global Minmax *k*-means ($p=0.3,\beta =0.3$)	*87*.*1170*	329.5055
Global Minmax *k*-means ($p=0.3,\beta =0.1$)	*87*.*1170*	329.5055
Global Minmax *k*-means ($p=0.3,\beta =0$)	*87*.*1170*	329.5055
Global Minmax *k*-means ($p=0.1,\beta =0.3$)	88.5673	329.4616
Global Minmax *k*-means ($p=0.1,\beta =0.1$)	88.5673	329.4616
Global Minmax *k*-means ($p=0.1,\beta =0$)	88.5673	329.4616
Global Minmax *k*-means ($p=0,\beta =0$)	90.8431	*329*.*4133*

Italic values indicate the best results in all the present results

**Table 5:** Comparative results on $S_4$ data set

Method	$E_{max}$	$E_{sum}$
*k*-Means	68.0815	110.6536
Global *k*-means	62.5878	*105*.*5999*
MinMax *k*-means ($p=0.5,\beta =0.3$)	*54*.*0427*	109.0927
MinMax *k*-means ($p=0.5,\beta =0.1$)	*54*.*0427*	109.0927
MinMax *k*-means ($p=0.5,\beta =0$)	54.0464	109.1226
MinMax *k*-means ($p=0.3,\beta =0.3$)	57.3660	106.6937
MinMax *k*-means ($p=0.3,\beta =0.1$)	57.3660	106.6937
MinMax *k*-means ($p=0.3,\beta =0$)	57.3660	106.6937
MinMax *k*-means ($p=0.1,\beta =0.3$)	61.0903	105.6490
MinMax *k*-means ($p=0.1,\beta =0.1$)	61.0903	105.6490
MinMax *k*-means ($p=0.1,\beta =0$)	61.0903	105.6490
MinMax *k*-means ($p=0,\beta =0$)	68.0815	110.6536
Global Minmax *k*-means ($p=0.5,\beta =0.3$)	*54*.*0427*	109.0927
Global Minmax *k*-means ($p=0.5,\beta =0.1$)	54.0464	109.1226
Global Minmax *k*-means ($p=0.5,\beta =0$)	54.0464	109.1226
Global Minmax *k*-means ($p=0.3,\beta =0.3$)	57.3660	106.6937
Global Minmax *k*-means ($p=0.3,\beta =0.1$)	57.3660	106.6937
Global Minmax *k*-means ($p=0.3,\beta =0$)	57.3660	106.6937
Global Minmax *k*-means ($p=0.1,\beta =0.3$)	61.0903	105.6490
Global Minmax *k*-means ($p=0.1,\beta =0.1$)	61.0903	105.6490
Global Minmax *k*-means ($p=0.1,\beta =0$)	61.0903	105.6490
Global Minmax *k*-means ($p=0,\beta =0$)	62.5878	*105*.*5999*

Italic values indicate the best results in all the present results

**Table 6:** The brief description of the real data sets

Data set	Instances	Attributes	Classes	Balanced
Coil2	216	1000	3	Yes
Iris	150	4	3	Yes
Seeds	210	7	3	Yes
Yeast	1350	8	5	No
Pendigits	10,992	16	10	Almost
User knowledge modeling	403	6	4	No

**Table 7:** Comparative results on the Coil2 data set

Method	$E_{max}$	$E_{sum}$
*k*-Means	79.0141	155.6635
Global *k*-means	105.2087	154.8112
MinMax *k*-means ($p=0.5,\beta =0.3$)	58.7115	154.6850
MinMax *k*-means ($p=0.5,\beta =0.1$)	57.1880	155.1839
MinMax *k*-means ($p=0.5,\beta =0$)	58.7317	154.5164
MinMax *k*-means ($p=0.4,\beta =0.3$)	58.8274	154.5812
MinMax *k*-means ($p=0.4,\beta =0.1$)	58.8519	154.5189
MinMax *k*-means ($p=0.4,\beta =0$)	58.8205	154.4097
MinMax *k*-means ($p=0.3,\beta =0.3$)	58.9824	154.5769
MinMax *k*-means ($p=0.3,\beta =0.1$)	58.9544	154.5170
MinMax *k*-means ($p=0.3,\beta =0$)	58.9147	154.4083
MinMax *k*-means ($p=0.2,\beta =0$)	59.1028	*154*.*4047*
MinMax *k*-means ($p=0.1,\beta =0$)	68.6188	154.6814
Global Minmax *k*-means ($p=0.5,\beta =0.3$)	*56*.*9899*	157.7988
Global Minmax *k*-means ($p=0.5,\beta =0.1$)	*56*.*9899*	157.7988
Global Minmax *k*-means ($p=0.5,\beta =0$)	57.7296	157.4811
Global Minmax *k*-means ($p=0.3,\beta =0.3$)	60.5913	157.1706
Global Minmax *k*-means ($p=0.3,\beta =0.1$)	60.8388	157.3204
Global Minmax *k*-means ($p=0.3,\beta =0$)	60.8388	157.3204
Global Minmax *k*-means ($p=0.05,\beta =0.3$)	102.5301	154.7850
Global Minmax *k*-means ($p=0.05,\beta =0.1$)	102.5301	154.7850
Global Minmax *k*-means ($p=0.05,\beta =0$)	102.5301	154.7850
Global Minmax *k*-means ($p=0.02,\beta =0.3$)	103.4904	154.7737
Global Minmax *k*-means ($p=0.02,\beta =0$)	103.4904	154.7737

Italic values indicate the best results in all the present results

**Table 8:** Comparative results on the Iris data set

Method	$E_{max}$	$E_{sum}$
*k*-Means	67.3007	147.2335
Global *k*-means	57.1672	139.9622
MinMax *k*-means ($\beta =0.3$)	47.4502	138.8884
MinMax *k*-means ($\beta =0.1$)	47.4502	138.8884
MinMax *k*-means ($\beta =0$)	47.4502	138.8884
Global Minmax *k*-means ($\beta =0.3$)	47.4502	138.8884
Global Minmax *k*-means ($\beta =0.1$)	47.4502	138.8884
Global Minmax *k*-means ($\beta =0$)	47.4502	138.8884

**Table 9:** Comparative results on the Seeds data set

Method	$E_{max}$	$E_{sum}$
*k*-Means	151.0572	428.7954
global *k*-means	*144*.*5954*	*428*.*6082*
MinMax *k*-means ($p=0.5,\beta =0.3$)	*144*.*5954*	*428*.*6082*
MinMax *k*-means ($p=0.5,\beta =0.1$)	144.6353	428.7769
MinMax *k*-means ($p=0.5,\beta =0$)	144.6353	428.7769
MinMax *k*-means ($p=0.4,\beta =0.3$)	145.3806	428.6408
MinMax *k*-means ($p=0.4,\beta =0.1$)	145.3806	428.6408
MinMax *k*-means ($p=0.4,\beta =0$)	145.3806	428.6408
MinMax *k*-means ($p=0.3,\beta =0.3$)	145.3806	428.6408
MinMax *k*-means ($p=0.3,\beta =0.1$)	145.3806	428.6408
MinMax *k*-means ($p=0.3,\beta =0$)	145.3806	428.6408
Global Minmax *k*-means ($p=0.5,\beta =0.3$)	*144*.*5954*	*428*.*6082*
Global Minmax *k*-means ($p=0.5,\beta =0.1$)	144.6880	429.0006
Global Minmax *k*-means ($p=0.5,\beta =0$)	144.6880	429.0006
Global Minmax *k*-means ($p=0.4,\beta =0.3$)	146.4214	428.6840
Global Minmax *k*-means ($p=0.4,\beta =0.1$)	146.4214	428.6840
Global Minmax *k*-means ($p=0.4,\beta =0$)	146.4214	428.6840
Global Minmax *k*-means ($p=0.3,\beta =0.3$)	146.4214	428.6840
Global Minmax *k*-means ($p=0.3,\beta =0.1$)	146.4214	428.6840
Global Minmax *k*-means ($p=0.3,\beta =0$)	146.4214	428.6840

Italic values indicate the best results in all the present results

**Table 10:** Comparative results on the Yeast data set

Method	$E_{max}$	$E_{sum}$
*k*-Means	13.5325	51.4444
Global *k*-means	13.4129	*50*.*9959*
MinMax *k*-means ($p=0.5,\beta =0.3$)	14.2165	52.7943
MinMax *k*-means ($p=0.5,\beta =0.1$)	22.6182	59.2278
MinMax *k*-means ($p=0.5,\beta =0$)	12.6324	51.7455
MinMax *k*-means ($p=0.4,\beta =0.3$)	11.1771	51.4789
MinMax *k*-means ($p=0.4,\beta =0.1$)	17.5689	54.6692
MinMax *k*-means ($p=0.4,\beta =0$)	12.6495	51.7366
MinMax *k*-means ($p=0.3,\beta =0.3$)	11.3333	51.3884
MinMax *k*-means ($p=0.3,\beta =0.1$)	11.6825	51.4354
MinMax *k*-means ($p=0.3,\beta =0$)	12.5912	51.7159
MinMax *k*-means ($p=0.1,\beta =0.3$)	12.6833	51.4565
MinMax *k*-means ($p=0.1,\beta =0.1$)	12.6655	51.4575
MinMax *k*-means ($p=0.1,\beta =0$)	12.6351	51.4379
Global Minmax *k*-means ($p=0.5,\beta =0.3$)	11.1427	51.3872
Global Minmax *k*-means ($p=0.5,\beta =0.1$)	21.2196	64.6526
Global Minmax *k*-means ($p=0.5,\beta =0$)	17.1350	53.5700
Global Minmax *k*-means ($p=0.4,\beta =0.3$)	11.3387	51.3334
Global Minmax *k*-means ($p=0.4,\beta =0.1$)	*10*.*9260*	51.3190
Global Minmax *k*-means ($p=0.4,\beta =0$)	22.5238	53.2086
Global Minmax *k*-means ($p=0.3,\beta =0.3$)	11.8178	51.2643
Global Minmax *k*-means ($p=0.3,\beta =0.1$)	11.8837	51.2450
Global Minmax *k*-means ($p=0.3,\beta =0$)	22.5238	53.2086
Global Minmax *k*-means ($p=0.2,\beta =0.3$)	12.2198	51.1261
Global Minmax *k*-means ($p=0.2,\beta =0.1$)	12.2198	51.1261
Global Minmax *k*-means ($p=0.2,\beta =0$)	12.1166	51.1379
Global Minmax *k*-means ($p=0.1,\beta =0.3$)	16.0342	53.6899
Global Minmax *k*-means ($p=0.1,\beta =0.1$)	16.0342	53.6899
Global Minmax *k*-means ($p=0.1,\beta =0$)	16.0179	53.6955

Italic values indicate the best results in all the present results

**Table 11:** Comparative results on the Pendigit data set

Method	$E_{max}$	$E_{sum}$
*k*-Means	11,540	60,963
Global *k*-means	12,549	*59,643*
MinMax *k*-means ($p=0.5,\beta =0.3$)	8510	62,094
MinMax *k*-means ($p=0.5,\beta =0.1$)	16,826	71,546
MinMax *k*-means ($p=0.5,\beta =0$)	7744	61,116
MinMax *k*-means ($p=0.4,\beta =0.3$)	7609	61,184
MinMax *k*-means ($p=0.4,\beta =0.1$)	10,394	63,285
MinMax *k*-means ($p=0.4,\beta =0$)	7740	61,100
MinMax *k*-means ($p=0.3,\beta =0.3$)	7948	60,993
MinMax *k*-means ($p=0.3,\beta =0.1$)	7918	60,993
MinMax *k*-means ($p=0.3,\beta =0$)	7924	60,994
MinMax *k*-means ($p=0.2,\beta =0.3$)	8854	60,825
MinMax *k*-means ($p=0.2,\beta =0.1$)	8824	60,823
MinMax *k*-means ($p=0.2,\beta =0$)	8854	60,825
MinMax *k*-means ($p=0.1,\beta =0.3$)	9630	60,753
MinMax *k*-means ($p=0.1,\beta =0.1$)	9611	60,759
MinMax *k*-means ($p=0.1,\beta =0$)	9630	60,753
MinMax *k*-means ($p=0.02,\beta =0.3$)	10,920	60,805
MinMax *k*-means ($p=0.02,\beta =0.1$)	10,919	60,805
MinMax *k*-means ($p=0.02,\beta =0$)	10,915	60,805
MinMax *k*-means ($p=0,\beta =0$)	11,539	60,962
Global Minmax *k*-means ($p=0.5,\beta =0.3$)	*6685*	60,394
Global Minmax *k*-means ($p=0.5,\beta =0.1$)	19,143	70,402
Global Minmax *k*-means ($p=0.5,\beta =0$)	6891	60,234
Global Minmax *k*-means ($p=0.4,\beta =0.3$)	6853	60,305
Global Minmax *k*-means ($p=0.4,\beta =0.1$)	6828	60,300
Global Minmax *k*-means ($p=0.4,\beta =0$)	6891	60,234
Global Minmax *k*-means ($p=0.3,\beta =0.3$)	6994	60,181
Global Minmax *k*-means ($p=0.3,\beta =0.1$)	6994	60,181
Global Minmax *k*-means ($p=0.3,\beta =0$)	6994	60,179
Global Minmax *k*-means ($p=0.2,\beta =0.3$)	10,860	59,918
Global Minmax *k*-means ($p=0.2,\beta =0.1$)	10,860	59,918
Global Minmax *k*-means ($p=0.2,\beta =0$)	10,860	59,918
Global Minmax *k*-means ($p=0.1,\beta =0$)	11,601	59,710
Global Minmax *k*-means ($p=0.02,\beta =0$)	12,330	59,645
Global Minmax *k*-means ($p=0,\beta =0$)	12,523	*59,643*

Italic values indicate the best results in all the present results

**Table 12:** Comparative results on the user knowledge modeling data set

Method	$E_{max}$	$E_{sum}$
*k*-Means	13.9469	41.6798
Global *k*-means	16.7506	41.2257
MinMax *k*-means ($p=0.5,\beta =0.3$)	11.1298	41.5906
MinMax *k*-means ($p=0.5,\beta =0.1$)	12.2885	42.2599
MinMax *k*-means ($p=0.5,\beta =0$)	11.3447	41.6220
MinMax *k*-means ($p=0.4,\beta =0.3$)	11.4587	41.5912
MinMax *k*-means ($p=0.4,\beta =0.1$)	11.4362	41.5951
MinMax *k*-means ($p=0.4,\beta =0$)	11.4776	41.5757
MinMax *k*-means ($p=0.3,\beta =0.3$)	11.8978	41.5361
MinMax *k*-means($p=0.3,\beta =0.1$)	11.8994	41.5463
MinMax *k*-means ($p=0.3,\beta =0$)	11.9395	41.5356
MinMax *k*-means ($p=0.2,\beta =0.3$)	12.5516	41.5503
MinMax *k*-means ($p=0.2,\beta =0.1$)	12.5544	41.5626
MinMax *k*-means ($p=0.2,\beta =0$)	12.5672	41.5508
Global Minmax *k*-means ($p=0.5,\beta =0.3$)	*10*.*9221*	41.2507
Global Minmax *k*-means ($p=0.5,\beta =0.1$)	*10*.*9221*	41.2507
Global Minmax *k*-means ($p=0.5,\beta =0$)	*10*.*9221*	41.2507
Global Minmax *k*-means ($p=0.4,\beta =0.3$)	11.0574	41.1979
Global Minmax *k*-means ($p=0.4,\beta =0.1$)	11.0574	41.1979
Global Minmax *k*-means ($p=0.4,\beta =0$)	11.0574	41.1979
Global Minmax *k*-means ($p=0.3,\beta =0.3$)	11.6460	41.0866
Global Minmax *k*-means ($p=0.3,\beta =0.1$)	11.6460	41.0866
Global Minmax *k*-means ($p=0.3,\beta =0$)	11.6460	41.0866
Global Minmax *k*-means ($p=0.2,\beta =0.3$)	11.8169	*41*.*0594*
Global Minmax *k*-means ($p=0.2,\beta =0.1$)	11.8169	*41*.*0594*
Global Minmax *k*-means ($p=0.2,\beta =0$)	11.8169	*41*.*0594*
Global Minmax *k*-means ($p=0.1,\beta =0$)	11.8169	*41*.*0594*
Global Minmax *k*-means ($p=0,\beta =0$)	14.9083	41.4720

Italic values indicate the best results in all the present results

In the experiment, we find the memory parameter $\beta$ and exponent parameter *p* affect the results in the MinMax *k*-means and the global Minmax *k*-means algorithm, and the variation does not have any rule. The practical framework that extends the MinMax *k*-means to automatically adapt the exponent to the data set proposed in Tzortzis and Likas (2014). They thought if the $p_{max}$ has been set, the programme can reach the lowest $E_{max}$ at $p\in [p_{init},p_{max}]$. However, our experiments show that it is not always correct. In Tables 10 and 11, when we set $p_{max}=0.3$, the results is better than $p_{max}=0.5$. In the experiment, it is easy to show that $E_{max}$ and $E_{sum}$ can not attain the lowest value at a time.

## Conclusions

We modified the global *k*-means algorithm to circumvent the singleton clusters. We also have presented the global Minmax *k*-means algorithm, with constitutes a deterministic clustering method in terms of the MinMax *k*-means clustering error i.e. minimize the maximum intra-cluster error. The method is independent of any starting conditions and compares favorably to the *k*-means algorithm and the MinMax *k*-means algorithm with multiple random restarts. We compare our method with the global *k*-means algorithm, too. The results of experiments show the advantage come together with the global *k*-means and the MinMax *k*-means algorithm i.e. we get a deterministic clustering method and need not any restart and our proposed algorithm always performs well.

As for future work, we plan to study in adapt method to determine the exponent parameter *p* and the memory parameter $\beta$, such that $E_{max}$ or $E_{sum}$ attain the lowest. And it would be better for us to tackling the two parameters at one time.
